# Femicide Fatal Risk Factors: A Last Decade Comparison between Italian Victims of Femicide by Age Groups

**DOI:** 10.3390/ijerph17217953

**Published:** 2020-10-29

**Authors:** Anna Sorrentino, Chiara Guida, Vincenza Cinquegrana, Anna Costanza Baldry

**Affiliations:** Department of Psychology, University of Campania ‘Luigi Vanvitelli’, 81100 Caserta, Italy; chiara.guida.1993@gmail.com (C.G.); vincenza.cinquegrana@unicampania.it (V.C.); annacostanza.baldry@gmail.com (A.C.B.)

**Keywords:** femicide, archive research, lethal risk factors, age-based differences

## Abstract

Femicide is a wide-spread lethal form of violence against women. Despite its diffusion, to date, very few studies analyzed possible victims’ age differences in regard to fatal risk factors for femicide. To this aim, we carried out archive research on Italian femicide cases in the last decade, by comparing prior types of violence suffered and motives for femicide, which are considered crucial fatal risk factors for femicide, across adolescent/young (15–24 years), adults (25–64 years) and older women (65–93 years). From 2010 to 2019 we found 1207 female victims. Characteristics of victims, perpetrators, and their relationship were consistent with those found by international studies and underlined that the majority of femicides were perpetrated by an intimate partner. The results regarding fatal risk factors comparisons across age groups showed the existence of significant differences regarding both types of violence suffered prior to femicide and motives for femicide. The results are discussed in terms of policy implication and intervention.

## 1. Introduction

Gender-based violence is a multifaceted phenomenon and represents a serious violation of women’s fundamental rights and freedom [1]. Discrimination, abuse, physical and sexual violence, genital mutilation, forced abortions, harassment, threats, gaslighting, deprivation of both public and private liberty, are considered among the ways through which violence against women can be carried out [2]. Worldwide, it is estimated that 1 in 3 women (35.6%) has been a victim of violence at least once in her life, mostly by an intimate partner [3,4,5]. In Italy, 6 million and 788 thousand (31.5%) women between 16 and 70 years, reported suffering from physical or sexual violence during their life: of these, 26.4% suffer these abuses from their current partner and 46.1% from the ex-partner [6].

Numerous studies pointed out the several negative physical (abdominal, thoracic, and brain injuries, fractures and lacerations, problems to reproductive health and chronic diseases such as asthma, cardiovascular disorders, diabetes, cancer, and neurological disorders) and psychological consequences (stress disorders, anxiety, depression, eating disorders, alcohol and drug abuse, sleep and somatoform disorders) of gender-based violence [4,7,8,9,10,11,12]. The fatal outcome of gender-based violence in many cases is the murder of a woman caused by a man, defined as “femicide,” “feminicide,” “gender-related homicide” [13], or “intimate partner homicide” [5]. With reference to 2017, 87 thousand women were killed worldwide intentionally [13]. Of the 87,000 femicides reported worldwide in 2017, 34.4% were caused by an intimate partner, 24.0% by family members, and 42.0% by perpetrators outside the family (friends, colleagues, acquaintances, and unknown) [13].

If we consider Italian victims, national statistic investigations highlighted that every year on average 155 women were victims of femicide, an incidence of one femicide every 2 and a half days [6,14].

In the past decade, the introduction of the Istanbul Convention on preventing and combating violence against women and domestic violence [1], increased social, political, and public interest and raised awareness on femicide, causing the introduction of specific laws to protect victims and prevent women’s homicides in countries like Italy (i.e., Law n° 119/2013, known as the “Law on Femicide”) [15]. Despite the increasing number of studies, reports, and data on femicide, to date, there is no universally recognized definition of this phenomenon and different studies adopt different definitions thus making it impossible to compare the data [16]. In addition to this, the variety of data collection methods between different countries and the absence of such data in some countries hampered any preventive efforts [9,17,18,19,20,21,22]. In fact, some studies adopted the definition introduced in 1977 by Diana Russell [23], conceptualizing femicide as a crime against women by male perpetrators, otherwise, other studies used the term feminicide [24] in order to highlight the societal and institutional responsibility for these women’s murder, while in some countries, no studies are available because the term does not exist [18,19,20].

For the present study we will adopt, according to the Vienna Declaration, the definition of femicide as “the killing of women and girls because of their gender” [25] (p. 2), considering all women and girls victims regardless of the perpetrator’s gender.

The high prevalence of femicide has generated attention not only among social activists and public opinion but also among scholars who, over the last 30 years, have invested great efforts to understand the underlying causes and fatal risk factors associated with femicide. Research has established that physical intimate partner violence has been found to be a crucial precursor of intimate partner femicide (IPF) [26,27]. In addition, jealousy, the woman’s desire to leave the man, and control seem to be the most common motives for intimate partner femicide [28].

Looking at the international literature on femicide we found that many studies focused on intimate partner homicide diffusion considering victims and/or perpetrators risk factors, and/or the contextual characteristic of the homicide [26,27,29,30], while other compared women-only victims of intimate partner violence (IPV) and victims of intimate partner homicide (IPH) in order to investigate fatal risk factors for femicide [31,32,33]. Despite the increasing number of studies on femicide, several issues still exist because not many studies have investigated femicide fatal risk factors by focusing on women’s age range.

Some studies analyzed adolescent/young femicides [34,35,36,37,38], even less considered older victims of femicide [39,40,41], and only one study compared adult and older victims of femicide [42]. Studies analyzing fatal risk factors for adolescent/young femicides were mainly descriptive, the majority of them were carried out in the U.S. and due to the different methodologies and the different age ranges considered to categorize adolescent/young victims of femicide, comparison of results is not always possible. However, all of them investigated motives for adolescent/young femicides, underlying that the most common were jealousy and controlling behaviors [32,33,34,35,36,37], arguments [34,35,38], and the end of the relationship [35,36,38]. Two studies investigated as possible fatal risk factors for adolescent/young femicides, previous violent behaviors suffered by the victims [34,36], one study investigated victims’ experiences of IPV and sexual abuse [35], and one study analyzed as possible fatal risk factors for young/adolescent femicides some types of violence such as threats, abuse during pregnancy and strangulation, however, none of the types of violent behaviors considered was significantly associated with the victims’ murder [37].

With regard to studies on older victims of homicide, some consider in general domestic homicide risk factors, including IPH (e.g., [43,44]), while one study investigated intimate partner homicide/suicide comparing perpetrators primary intent across the young, middle, and elder adults [41]. The results of these studies showed that significant fatal risk factors for femicide among older people were mental illness or breakdown and mercy killing [39,40,41,44]. The only study investigating age-based risk factors for femicide compared only adult and older victims with regard to victims’ marital status, cause of death, weapon, motives for femicide, founding that older victims were more likely to be killed for felony murder [42].

However, the majority of studies looked at femicide characteristics of women between 25 and 65 years [5], underlying that relevant relational fatal risk factors for femicide were the previous history of IPV, including stalking, threats and controlling behaviors, jealousy, and arguments [26,27,29,30,33,45].

Investigating possible differences across adolescent/young, adult, and older victims in terms of fatal risk factors for femicide could be crucial in terms of prevention and intervention strategies, as underlined the peak age of femicide is from 30 to 49 years [45,46,47].

Therefore, categorizing victims of femicide according to recent international literature suggestions [25,48,49], we aimed to compare adolescent/young, adult and older victims of femicide occurred in Italy with regard to two relevant fatal risk factors that are types of violence suffered and motives of femicide, considering a time span of a decade, from 2010 to 2019. This time span was adopted as our reporting period to account for the several legislative reforms in Italy, from the Law n° 38/2009 regulating stalking crimes by way of Italy’s ratification of the Istanbul Convention [1] (Law n° 119/2013, known as the “Law on femicide”), to the recent Law n° 69 of 19 July 2019 (known as the ‘Red Code’) (which, introduced a series of new offenses, such as forced marriage, the deformation of an individual’s appearance through permanent facial injuries and the unlawful dissemination of sexually explicit images or videos or revenge porn. Moreover, the ‘Red Code’ strengthened the sanctions for the crimes of stalking, sexual violence, and domestic violence and increased the applicable sanctions for aggravated circumstances.), that introduced further measures to increase the effectiveness of judicial responses to violence against women and improve victims’ protection.

As far as we know, this is the first archive study carried out considering Italian cases on femicide covering a 10-year period, during which as underlined above, and due to several political, legislative and social changes, the public and mass-media interest in femicide has grown [15].

## 2. Method and Procedure

The present research was a preliminary archive research study useful to the further implementation of a wider study on femicides called FATHERS (Female Abuse, Threats and Homicide: Emotional Responses and Screening), that was approved by the Departmental Ethics Committee (18/2016 on the 27 September 2016).

Information and data were gathered about all cases of female subjects killed from January 2010 to December 2019 in Italy; according to the definition of femicide, adopted for the purpose of the present study all girls and women killed because of their gender were included in our sample [25]. Information and characteristics of femicide cases happened in Italy from 2010 to 2019 were searched by consulting databases of local, regional, and national online media and newspapers; those were the most suitable sources for collecting our data of interest and were able to provide us with as much information as we needed on the women [18]. A total of 3092 online articles were consulted, using the following keywords: “female homicide,” “feminicide,” “woman killed,” “wife homicide,” “girlfriend homicide,” “woman murdered,” “domestic homicide.” Each case of femicide identified was monitored over time, to have updated and more detailed information about the murder and the proceedings. Information collected about femicides in Italy from 2010 to 2019 were codified according to a Fatality Review Protocol developed for the purpose of the present study, considering the COST Action Guidelines [18] (The European Union program entitled “European Cooperation in Science and Technology” (COST) launched the “Femicide across Europe” COST Action in 2013, establishing a European transnational cooperation among experts addressing about femicide.). The fatality review protocol included 26 categories, and six macro thematic Areas: (1) Temporal Data (year, day, month); (2) Geographical data (region, province, municipality); (3) Characteristics of the victims (e.g., name and surname, age, nationality, occupation); (4) Characteristics of the author (e.g., name and surname, gender, age, nationality, occupation, previous crimes, use of psychoactive substances); (5) Characteristics related to the relationship context of the author and the victim (e.g., type of relationship, presence/absence of children, children in common/not in common, previous violence); (6) Femicide characteristics (e.g., type of crime, type of weapon, motives, and place of murder).

For the purpose of the present study, the following set of variables were analyzed:

- Characteristics of the victims included socio-demographic information such as age, nationality, occupation, and presence of children
- Characteristics of the perpetrator included information about gender, age, nationality, occupation, previous crimes, and use of psychoactive substances
- Characteristics related to the relationship context of the author and the victim included the type of relationship, presence of children both common or of only the victim or the perpetrator, previous violence, previous types of violence suffered by victims (threats, stalking, physical violence, sexual violence and controlling behaviors) and motives for femicide (jealousy, incapability to accept the end of the relationship, quarrels and conflicts, victims and/or perpetrators’ mental and physical illness).

### Data Analyses

The data collected within the database were analyzed using the SPSS statistical package (version 21.0, IBM Milano, Milan, Italy) and MAKESENS application [49].

Descriptive statistics were carried out for each of the aforementioned categories. In order to analyze possible differences among victims of femicide, we divided women into three age groups, according to other studies in the literature [48,50], that consider women aged from 15 to 24 years old as “adolescent/young women,” from 25 to 64 years as “adult women” and from 65 to 93 as “older women.”

We assessed the significance of annual trends in femicide using the Mann–Kendall trend test [51,52], To examine the relationship between women’s age ranges (“adolescent/young women,” “adult women,” “older women”) and all the different types of violence suffered included in the study (“physical violence,” “threats,” “sexual violence,” “stalking” and “controlling behaviors”), the adjusted standardized residuals (ASR) were calculated to test the significance of the difference between the observed frequencies and the actual frequencies in each cell, indicating whether cell numbers differed from chance expectation [53]. Finally, motives of femicides were compared for each women’s age range against all others by using odds ratios (OR). We assessed statistical significance at least at 0.05 level for all the statistical analyses performed.

## 3. Results

Our sample was composed of a total of 1.207 female victims of femicide in Italy, aged between 15 to 93 years (*M =* 50.41, *SD* = 19.70). 47.0% of female homicides were committed in the North, followed by the South (34.7%) and the Center (18.3%) of Italy.

**Figure 1:**
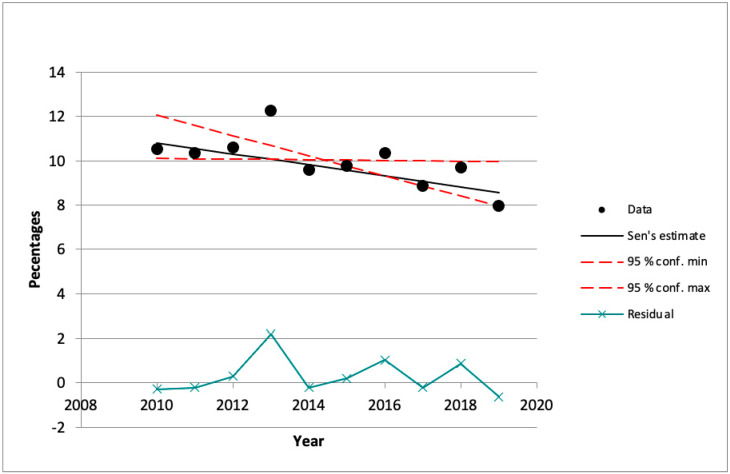
Changes in Italian femicides cases over time.

With regard to femicides prevalence in the last decade, on average about 121 (*SD* = 14) women were killed per year. Every month about ten women lost their life. Analyzing femicides prevalence across years, we found that the highest number of female murders was recorded in 2013 (12.2%). The results of the Mann–Kendall trend test showed a statistically significant decreasing trend in femicides over time (Z = −2.07, Q= 2.49E − 01, *p* = 0.039). The Sen’s slope estimates indicated abrupt increases in femicides in 2013 and 2016 and a slight decrease in 2019 (see Figure 1).

### 3.1. Characteristics of Victims by Age

Respectively 8.1%, 64.8%, and 27.1% of victims were adolescent/young, adult, and older. With regard to the geographical area, it seems that independently by the victims’ age groups, the majority of femicides were committed in the North of Italy.

More than 70.0% of adult and older victims of femicide were Italian, while slightly less than half of adolescent/young victims were foreign.

With regard to victims’ occupation 57.1% of adolescent/young victims were unemployed, 42.7% of adult women were in low specialization jobs (e.g., caregiver, waitress, hairdresser), 27.9% were in high specialization occupations (e.g., teacher, manager, employee) and 82.7% of older women were retired. Only 32.6% of adolescent/young women had at least one child (for more detail see Table 1).

### 3.2. Characteristics of Perpetrators by Age

From a total number of 1147 of offenders aged between 15 and 97 years (*M =* 48.45 *SD* = 17.21), 98.9% were male, 1.1% were female (see Table 2).

Regardless of the age group considered, it seems that most of the perpetrators were Italian. With regard to perpetrators’ occupation, 56.0% of adolescent/young authors were in low specialization occupations (e.g., workers in companies, plumbers, electricians, farmers or crafts), 12.6% of adult authors were members of law enforcement, and 68.9% of older perpetrators were retired.

18.3% of perpetrators had committed previous crimes (e.g., crimes against property, drug dealing or crimes against individuals), of these respectively 12.8%, 22.5%, and 5.7% were committed by adolescent/young, adults and older perpetrators. 15.7% of perpetrators used some psychotropic substances such as alcohol, cocaine, or marijuana, with adult authors reporting the highest percentage of usage of these substances (18.2%).

As expected, older perpetrator registered the highest percentages of physical and/or mental disorders (24.2%), followed by adult authors (9.2%).

Finally, respectively 3.7%, 4.8%, and 0.7% of adolescent/young, adult and older perpetrators had acted violence in previous relationships.

### 3.3. Characteristics of the Relationship

In about 60.3% of femicides, the perpetrator was an intimate partner or ex-partner (33.8% husbands, 3.6%, boyfriends, 6.8% cohabitants, 6.9% ex-husbands, 5.8% ex-boyfriends, or 3.4% ex-cohabitants); in 13.7% of the cases, the author was a close family member (i.e., in 10.2% of cases, the author turns out to be the victim’s child). The remaining 26.0% includes perpetrators outside the family.

69.1% of the victims were mothers, of these 34.1% had a child with the perpetrator.

25.4% of the women had already suffered violence from the author: for 55.2% of those, it was physical and psychological violence and to a lesser extent physical violence (10.8%) or physical and psychological violence plus stalking (9.8%). Furthermore, 15.7% were victims of stalking, 4.5% had been seriously threatened and 2.4% were victims of sexual violence.

Regarding motives for the femicide, the inability to accept the end of the relationship from the author is the most frequent (23.3%); following this, in 15.6% of the cases the femicide was the result of jealousy between the partners, and in 14.4% of cases the femicide happened in an escalation of violence started because of quarrels or conflicts in the couple. Finally, respectively 12.9% and 7.2% of the motives for femicides were due to the presence of physical or mental illness of the author or the victim.

### 3.4. Types of Violence across Women’s Age Groups

Firstly, we analyzed the diffusion of different types of violent behaviors experienced by victims from their perpetrator (see Table 3). Controlling behaviors were the most common type of violence suffered by victims regardless of the age group considered, followed by stalking among adolescent/young and adult women and by physical violence among older victims.

Older victims did not experience sexual abuse and threats differently from the other two groups.

Then, we examined the existence of possible significant age ranges differences across victims with regard to the different types of violent behaviors suffered.

The results showed that adolescent/young women significantly differed from adult women and older women [χ^2^_(2)_ = 6.03, *p* = 0.049] in terms of stalking suffered indicating that adolescent/young women were significantly stalked more than the other two age groups (ASR = 2.4). No significant differences emerged between the other types of violent behaviors experienced and women’s age.

### 3.5. Motives for Adolescent/Young, Adult and Older Women’s Femicide

Subsequently, motives for the femicide were compared for each women’s age range against all others by calculating odds ratios. Table 4 compares motives for femicides for each age range against the rest. Concerning the dimension of jealousy, adult women were more likely than adolescent/young and older women to have been a victim of femicide (OR = 4.23, 95% CI (2.65–6.75), *p* < 0.001). Adult women were also more likely to be killed by the perpetrator with the motive of “incapability to accept the end of the relationship” and femicides that happen as an escalation of quarrels and conflicts than the other two groups (OR = 4.73, 95% CI (3.12–7.17), *p* < 0.001). On the other hand, older women were almost ten times more likely to be killed when there was the presence of a physical or mental illness, either in the perpetrator or the victim (OR = 10.88, 95% CI (5.50–21.51), *p* < 0.001).

## 4. Discussion and Conclusions

Femicide is the lethal outcome of violence against women [5,13] and a worldwide phenomenon. Despite the incidence of women’s homicide, and the fact that a significant number of women were killed by their partner or former partners, still few studies have been carried out in order to identify victims of femicide lethal risk factors [27,29,30,31,32,33]. In particular, very few studies have investigated fatal risk factors considering victims’ age range [41,42,43], and as far as we know, no studies compared victims’ fatal risk factors for femicide by considering three different age groups, that is adolescent/young, adult and older women, by carrying out an archive study covering a ten-year period of femicides in Italy in order to evaluate also possible changes in women’s killings over time.

From 2010 to 2019 we found 1207 femicide cases in Italy, noting a significant decreasing trend over time in the percentages of annual victims of femicide. This downward trend seems to start from 2013, which had been a crucial year for Italy, as this year corresponds both to the highest number of femicides recorded and the ratification of the Istanbul Convention [1] in Italy. Based on our results we could hypothesize that 2013 represented a milestone for the Italian governmental, political and social movements and parties, after which Law n° 119/2013 and Law n° 69/2019 were introduced by the Italian Parliament. Even if to date no data on the effectiveness of these legislative measures in terms of prevention and reduction of femicide rates are available, the increased media and social media attention to femicide could have raised public awareness, as underlined by a recent report according to which more than 90% of Italians consider IPV as not acceptable [54]. Consistent with the international literature, we found that the majority of women’s femicide (60.3%) were perpetrated by an intimate partner [4,5,13,34,36,37,38].

In addition, the results related to victims’ and perpetrators’ characteristics and their relationship were consistent with risk factors known in international research [5,13,26,27,28]. In consideration of this, and taking into account that femicide is transversal across all age groups, we focused on a particular and little investigated aspect related to femicide, that is victims’ age, in order to assess possible age groups differences with regard to the two main lethal risk factors described in the literature, which are previous violence suffered [26,27] and motives for femicide [28,32,33,34,35,36,37,38].

As expected, our results underlined the existence of significant differences across victims’ age groups. Regardless of victims’ age, all types of violent behaviors considered were quite spread in our sample. Similar to other studies [26,29,30,55], the most common type of violent behaviors experienced by victims were controlling behaviors, followed by stalking for adult women and physical violence for older victims. In particular, adolescent/young women reported higher levels of stalking and controlling behaviors suffered than the other two groups of women considered. These results seem to highlight and confirm that for young and adolescent victims such forms of controlling and intrusive behaviors are significant lethal risk factors [34,35,37].

With regard to the second fatal risk factor investigated, which is motives for femicide, our results confirmed that among adult victims the major motives for the women’s killing were jealousy, incapability to accept the end of the relationship, and quarrels and conflicts [26,27,29,56,57,58,59], indicating also that compared to the other victims, the presence of such motives seems to significantly increase adult women’s lethal risk. Differently, older women compared to adolescent/young and adult women were significantly more likely to be killed due to the victim and/or perpetrator’s mental and/or physical illness.

The investigation of such aspects could have several prevention and intervention implications. Our results, consistent with other studies, underlined the need to develop primary and secondary prevention activities and the need for further studies on lethal risk factors across victims’ age groups. This in terms of primary prevention, means the necessity to develop actuarial self-assessment instruments age-matched and assess their efficacy in terms of increased awareness and risk recognition. The development of these instruments jointly with other primary prevention activities targeting children and adolescents, aimed to reject violence and recognize risky behaviors, should result in the early recognition of some underestimated and normalized behaviors often considered as simply as gestures of love [37]. Prediction of revictimization has become a major research focus in the area of intimate partner violence, and thus, understanding factors that are associated with revictimization and fatal event have crucial implications, for both professionals and women, in helping them to maximize their ability to adopt preventive measures accordingly [60]. Furthermore, in terms of secondary prevention activities, the presence of stalking and controlling behaviors in adolescent/young women, could be considered as significant and lethal risk factors for this age groups’ femicide, thus leading to an adequate risk assessment by professionals and to the activation of targeted and rapid risk management protocols.

With regard to motives of femicide, our results underlined the existence of significant differences between adolescent/young, adult and older women, supporting the need for further studies in order to investigate and confirm the existence of such differences in lethal risk factors among victims’ age groups. In particular, consistent with international literature jealousy, quarrels and conflicts and the perpetrator’s incapability to accept the end of the relationship are all motives that significantly increase adult victims’ risk of lethal violence. The presence of the victim and/or perpetrator’s physical and mental illness were found to be significant lethal risk factors, increasing the odds for femicide among older victims. Consistent with other studies [39,40,41,44], we can hypothesize that in fact these women have been often forgotten in the research on femicide. Several possible explanations of motives for femicide among older women could be found in both cultural and educational context since some of these femicides could be the fatal result of a long history of violence never recognized nor reported or, on the contrary, they could have a salvific purpose, “as a solution to deteriorating health situations” [39] (p. 25), that underline the possible need to increase older people’s social support levels and reduce their sense of isolation.

The current study has certain limitations. As in the majority of femicide studies, information about the event, the victim, the perpetrator, and their relationship were collected by searching and reading online newspaper articles [57]. Another possible limitation of the present study could be represented by the absence of the so-called “control group” or “abused women” [57,61] to compare lethal and non-lethal risk factors across groups.

Despite the aforementioned limitations, to the best of our knowledge, the present study is the first one aimed at investigating femicide’s risk factors across different age groups.

Further studies should better investigate lethal and non-lethal risk factors for femicide by considering victims age group differences, and by comparing abused women and victims of femicide, in order to design, implement and assess the effectiveness of prevention and intervention programs and age-based activities in terms of women protection and reduction of femicides.

## Figures and Tables

**Table 1:** Descriptive statistics for victims of femicide by age groups.

		Adolescent/Young Women	Adult Women	Old Women
Age		*M* = 20.91 (*SD* = 2.49)	*M* = 42.88 (*SD* = 10.34)	*M* = 77.24 (*SD* = 7.59)
Nationality	Italian	56.3%	70.2%	97.2%
	Foreign			
Femicide geographical area	North	40.8	47.1	48.6
	Centre	20.4	16.9	21.1
	South	38.8	36.1	30.3
Occupation	Unemployed	57.1	21.2	6.3
	Low specialization	37.5	42.7	6.8
	High specialization	5.4	27.9	4.2
	Retired	0.0%	8.2%	82.7%
Presence of children	Yes	32.6	71.3	76.1
	No	67.4	28.7	23.9

**Table 2:** Descriptive statistics for perpetrators by age groups.

		Adolescent/Young Perpetrators	Adult Perpetrators	Older Perpetrators
Age		*M* = 21.02 (*SD* = 2.24)	*M* = 44.23 (*SD* = 10.08)	*M*= 76.29 (*SD* = 7.33)
Nationality	Italian	71.1	78.4	98.6
	Foreign	28.9	21.6	1.4
Occupation	Unemployed	36.0	22.8	0.0
	Law enforcement	4.0	12.6	4.3
	Low specialization	56.0%	47.4	15.0
	High specialization	4.0%	12.9	11.8
	Retired	0.0%	4.3	68.9
Previous crimes	Yes	12.8	22.5	5.7
	No	87.2	77.5	94.3
Substance use	Yes	14.8	18.2	3.3
	No	85.2	81.8	96.7
Physical or mental disorders	Yes	3.6	9.2	24.2
	No	96.4	90.8	75.8
Previous violent relationships	Yes	3.7	4.8	0.7
	No	96.3	95.2	99.3

**Table 3:** Types of violence across women’s age groups.

		Physical Violence	Threats	Sexual Violence	Stalking	Controlling Behaviors	Total
Adolescent/young women	Observed	4	2	2	9	11	29
	Expected	3.1	1.3	.7	4.6	16.0	
	Ratio	1.29	1.53	2.85	1.95	0.68	
	Row %	13.8%	6.9%	6.9%	31.0%	37.9%	100.0%
	Column %	12.9%	15.4%	28.6%	20.0%	7.0%	10.1%
	Residual	0.9	0.7	1.3	4.4	−5.0	
	Standard Residual	0.5	0.6	1.5	2.1	−1.3	
	ASR	0.5	0.6	1.6	2.4 ^a,^*	−2.0	
Adult women	Observed	22	11	5	33	129	228
	Expected	24.7	10.4	5.6	35.9	126.0	
	Ratio	0.89	1.05	0.89	0.91	1.02	
	Row %	9.6%	4.8%	2.2%	14.5%	56.6%	100.0%
	Column %	71.0%	84.6%	71.4%	73.3%	81.6%	79.7%
	Residual	−2.7	0.6	−0.6	−2.9	3.0	
	Standard Residual	−0.5	0.2	−0.2	−0.5	0.3	
	ASR	−1.3	0.4	−0.6	−1.2	0.9	
Old women	Observed	5	0	0	3	18	29
	Expected	3.1	1.3	0.7	4.6	16.0	
	Ratio	1.61	0.00	0.00	0.65	1.12	
	Row %	17.2%	0.0%	0.0%	10.3%	62.1%	100.0%
	Column %	16.1%	0.0%	0.0%	6.7%	11.4%	10.1%
	Residual	1.9	−1.3	−0.7	−1.6	2.0	
	Standard Residual	1.0	−1.1	−0.8	−0.7	0.5	
	ASR	1.2	−1.2	−0.9	−0.8	0.8	
Total	Observed	31	13	7	45	158	286
	Row %	10.8%	4.5%	2.4%	15.7%	55.2%	100.0%
	Column %	100.0%	100.0%	100.0%	100.0%	1000.0%	100.0%
	Total %	10.8%	4.5%	2.4%	15.7%	55.2%	100.0%

Note: ^a^ χ^2^_(2)_ = 6.03, *p* = 0.049. * Refer to ASR > 1.96, or <−1.96, significant at *p* < 0.05.

**Table 4:** Comparison of motives of femicide for each age range against the rest.

		Jealousy	Incapability to Accept the End of the Relationship	Quarrels and Conflicts	Victim’ Mental and Physical Illness	Perpetrator’ Mental and Physical Illness
Adolescent/young women	%	12.4	11.5	1.9	1.3	2.2
	OR (CI)	1.31 (0.72–2.39)	1.20 (0.69–2.08)	0.18 ** (0.06–0.61)	0.12 * (0.02–0.90)	0.20 * (0.06–0.70)
Adult women	%	83.4	84.9	72.9	12.8	46.0
	OR (CI)	4.23 *** (2.65–6.75)	4.73 *** (3.12–7.17)	2.26 *** (1.48–3.45)	0.12 *** (0.06–0.25)	0.72 (0.48–1.08)
Older women	%	4.1	3.6	25.2	85.9	51.8
	OR (CI)	0.08 *** (0.04–0.17)	0.07 *** (0.03–0.13)	0.06 * (0.39–0.93)	10.88 *** (5.50–21.51)	1.92 ** (1.27–2.90)

Note: * *p* < 0.05; ** *p* < 0.01; *** *p* < 0.001. OR = Odds Ratio, CI = Confidence Interval.
